# Long-term room temperature preservation of corpse soft tissue: an approach for tissue sample storage

**DOI:** 10.1186/2041-2223-2-17

**Published:** 2011-08-16

**Authors:** Mariela Caputo, Luis A Bosio, Daniel Corach

**Affiliations:** 1 Servicio de Huellas Digitales Genéticas, Facultad de Farmacia y Bioquímica, Universidad de Buenos Aires, Buenos Aires, Argentina; 2 Servicio de Antropología Forense, Cátedra de Medicina Legal y Deontología Médica, Facultad de Medicina, Universidad de Buenos Aires, Buenos Aires, Argentina

## Abstract

**Background:**

Disaster victim identification (DVI) represents one of the most difficult challenges in forensic sciences, and subsequent DNA typing is essential. Collected samples for DNA-based human identification are usually stored at low temperature to halt the degradation processes of human remains. We have developed a simple and reliable procedure for soft tissue storage and preservation for DNA extraction. It ensures high quality DNA suitable for PCR-based DNA typing after at least 1 year of room temperature storage.

**Methods:**

Fragments of human psoas muscle were exposed to three different environmental conditions for diverse time periods at room temperature. Storage conditions included: (a) a preserving medium consisting of solid sodium chloride (salt), (b) no additional substances and (c) garden soil. DNA was extracted with proteinase K/SDS followed by organic solvent treatment and concentration by centrifugal filter devices. Quantification was carried out by real-time PCR using commercial kits. Short tandem repeat (STR) typing profiles were analysed with 'expert software'.

**Results:**

DNA quantities recovered from samples stored in salt were similar up to the complete storage time and underscored the effectiveness of the preservation method. It was possible to reliably and accurately type different genetic systems including autosomal STRs and mitochondrial and Y-chromosome haplogroups. Autosomal STR typing quality was evaluated by expert software, denoting high quality profiles from DNA samples obtained from corpse tissue stored in salt for up to 365 days.

**Conclusions:**

The procedure proposed herein is a cost efficient alternative for storage of human remains in challenging environmental areas, such as mass disaster locations, mass graves and exhumations. This technique should be considered as an additional method for sample storage when preservation of DNA integrity is required for PCR-based DNA typing.

## Background

Disaster victim identification (DVI) represents one of the most difficult challenges in forensic sciences. This complex task is usually approached within a multidisciplinary context [1-4]. In addition to the conventional identification criteria applied, including visual recognition, odontology, dactyloscopy and antemortem information, DNA-based identification represents, after 20 years of successful use, the gold standard for assisting in DVI [5]. Tissue preservation and subsequent DNA typing have become essential techniques, especially in disasters involving body fragmentation.

DNA purification from soft tissue is a widely used technique with higher yields than those obtained with bone samples, but soft tissue needs to undergo preservation procedures in order to prevent decay [6]. Cryopreservation is the procedure of choice to ensure long-term storage for such tissues; however, careless deep freezing of a sample will induce ice crystal formation inside the cell, affecting its membrane integrity [7]. Therefore, after thawing, the release of endogenous enzymes can degrade other cellular components [8]. Moreover, handling and preserving large numbers of samples requires a large volume of freezing equipment, which is not always available in disaster-challenged areas. Several specific methods for specimen preservation have been described, some of which exhibit adequate storage capacity for different tissues, for example, cryopreservation [7], formalin-fixed paraffin-embedded tissue, despite producing the disadvantage of irreversible changes in the molecular structure [9,11], salt water [10], alcohol-based tissue fixatives, dimethylsulfoxide (DMSO), commercial kits such as the Oragene DNA self-collection kit (DNA Genotek, Ottawa, Ontario, Canada) [11-14], LST buffer [12], and RNA*later *(Ambion, Austin, TX, USA) [13].

In this study, an efficient and economical procedure based on the use of dry table salt, mostly NaCl, is proposed and tested. It is based on rapid dehydration and modification of the ionic concentration of muscle tissues, such as those gathered from fragmentary human remains after mass fatalities. The method proposed is an alternative approach that offers advantages reflected in the DNA yields and quality of the genetic profiles obtained.

## Results

In order to test the efficiency and reliability of sodium chloride as a soft tissue preservation method leading to recovery of DNA samples suitable for polymorphic loci analysis, human psoas muscle was exposed to three different preservation conditions at different storage times over a 1-year period. Muscle samples of similar weight maintained at room temperature were exposed to solid sodium chloride (table salt), garden soil, or were left untreated. DNA was extracted and quantified, and autosomal, sex and mtDNA markers were analysed.

**Figure 1:**
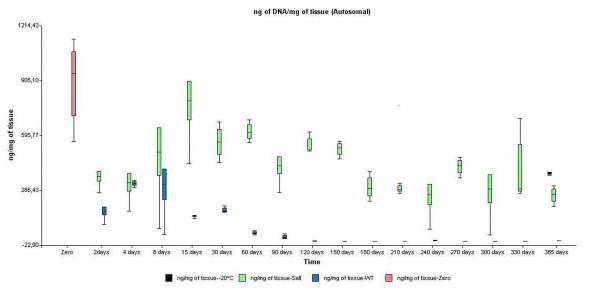
**Boxplot displaying DNA quantification results**. Autosomal DNA at room temperature with table salt (green), with out treatment (wt) (blue), -20°C 365 days (black) and 0 time (red).

Figure 1 shows the autosomal DNA quantification and displays boxplots of samples exposed to table salt or left untreated stored at room temperature. Since there was no DNA recovery after 2-day exposure to garden soil, the results were not plotted, though all the samples were purified and quantified. DNA quantities recovered from samples stored in salt were similar up to the complete storage time and underscored the effectiveness of the preservation method.

Haplogrouping of mitochondrial and Y-chromosomes was successful in all selected samples due to the high sensitivity of real-time PCR and the reduced amplicon length (data not shown).

**Figure 2:**
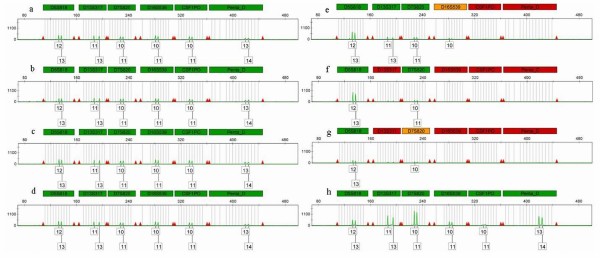
**Autosomal markers on green channel (PowerPlex16)**. Electropherograms of autosomal markers on green channel for PowerPlex16 panel for different samples. **(a) **Zero time; preserved with salt; **(b) **30 days; **(c) **120 days; **(d) **365 days. **(e) **Untreated; 30 days; **(f) **120 days; **(g) **365 days. **(h) **Control sample, 365 days at -20°C. To facilitate comparison, the scale is the same for all the electropherograms.

Figure 2 shows autosomal marker amplification on the green channel. The genotype quality status of all samples exposed to salt at room temperature was ascertained by the expert Genemapper-IDX software. A green flag showed a good quality of purified genetic material, whereas yellow or red flags in some markers after 30, 120 and 365 days in the untreated samples exposed to room temperature indicated DNA degradation. Male specific quantification by Plexor (see Materials and methods) showed no significant differences as expected.

After 1 year, visual inspection of the samples maintained in closed tubes containing NaCl under the test conditions showed that they were mostly dry and had kept the original colour and fibrous texture of the tissue. However, it was observed that those samples in tubes with garden soil exhibited a whitish colour compatible with fungal growth. Finally, the untreated samples kept in closed tubes revealed a dense fluid aspect, probably due to autolysis caused by long exposure time.

## Discussion

In some cases of forensic interest such as natural or human-induced mass fatalities, the predominantly available tissue type might be soft tissue. Accordingly, the development of an effective preservation method that prevents degradation is critical. Collected samples for DNA-based human identification are usually stored at -20°C or -70°C to halt the degradation processes of human bodies after death. However, it is essential to take the precaution of not thawing the material to prevent the action of endogenous enzyme release that might degrade DNA. In certain situations (for example, mass disasters), immediate refrigeration of samples is usually not possible. Rapid decomposition may occur within 24-48 h, so efficient methods for non-refrigerated corpse tissue preservation should be available [1-4].

An alternative preservation approach was developed in our study. DNA quantification of samples preserved in dry salt at room temperature at different storage times showed similar DNA yields. The deviation for each point could be due to two causes: the heterogeneous nature of the muscle and the intrinsic variability of DNA extraction and quantification methods. We compared the salt-based storage condition with untreated muscle tissue maintained at room temperature as a control for endogenous degradation, or exposed to garden soil as a control for environmental degradation.

As previously described, LST buffer (a combination of K and Na salts with detergents and sodium azide used as preservative) was used by Graham *et al*., who obtained approximately 1.0-16 ng/mg of tissue when collected samples ranged from approximately 5-100 mg of tissue stored in 1 ml LST buffer or stored in Oragene collection pots. The quality of the genetic profiles using AmpF*l*STR SGM Plus was comparable in all cases; 28 PCR cycles were performed to generate a partial profile in samples stored for 36 to 52 weeks. The authors concluded that LST buffer is a cost-effective alternative preservation solution for at least 4 weeks [12]. In addition, Fregeau and colleagues obtained high quality genetic profiles with DNA extracted from biopsies of human smooth muscle samples stored in 1 ml of GenoFix up to 365 days at room temperature or 3.5 years at -20°C (DNA Genotek) or paraffin-embedded tissues genotyped by AmpFlSTR COfiler and Profiler Plus [14]. Likewise, Mukaida *et al*. obtained DNA suitable for typing with AmpFlSTR Profiler starting with 10 mg of tissue collected from fragmentary human remains recovered from the ocean 2 days after a plane crash [10]. Additionally, Michaud *et al*. obtained suitable DNA starting from 0.2-0.5 g of pig tissue stored in a solution-based in DMSO, NaCl to saturation and ethylenediaminetetra-acetic acid (EDTA), although DNA quality evaluation did not include short tandem repeat (STR) analysis [13]. RNA*later *(Ambion), a commercial aqueous sulfate salt solution that rapidly fixes fresh tissue at room temperature, was studied as a preservation tissue solution. The principal disadvantage of this solution is the interference with DNA extraction as the organic and aqueous layers are inverted, which might interfere with routine laboratory work [13].

In our preservation method, the yields ranged 397.9 ± 131.5 ng of autosomal DNA/mg of tissue, starting from 200 mg ± 20 mg of tissue stored up to 365 days. DNA recovery was much higher than that described in the above-mentioned reports. The underlying principle of use of dry table salt (mostly NaCl) is the rapid dehydration produced by passive water extraction induced by the surrounding hygroscopic medium and the modification of intracellular ionic concentration caused by the passive intake of dissolved salt into the cells during the first steps of decomposition. Both in this case and in that of corpses retrieved from seawater, as well as in the solution proposed by Michaud *et al*., the high concentration of NaCl could account for tissue preservation as a result of a change of ionic concentration within the tissue [10,13] and desiccation. However, the latter effect produced by heat (oven-dried tissues) or the hygroscopic effect (silica-stored samples) required extra care during organic extraction, and DNA recovery efficiency was less effective than in other methods such as alcohol-based tissue fixative or different storage solutions [13,14]. Sodium chloride has been proposed in the recommendations for disaster victim identification [15,16], although its routine use is not generalised. However, since 1996, this approach has been successfully used in our laboratory for preserving corpse tissue obtained from exhumations as well as in DVI cases [17].

The preservation strategy tested herein is similar to that employed by the ancient Egyptians in their embalming process. It is known that artificial mummification allows the preservation of morphological features. Although there is no written record of Egyptian mummification processes, it is known that those procedures included evisceration of the corpse followed by desiccation with natron (sodium salts such as sodium carbonate/bicarbonate) without the application of organic preservatives. Consequently, it prevents both microbial attack associated with subsequent biological tissue degradation and loss of molecular structure [18,19].

With regard to the material exposed to garden soil, the expected fast DNA degradation due to microbial action was observed. The quantification method uses an internal PCR control (IPC), which indicated the presence of inhibition substances. In the case of soil samples, all of them were inhibited, which could be explained by the presence of humic acid content. Humic acid is known to inhibit commercial Taq polymerases to variable degrees and it can decrease DNA-DNA interaction interfering with primer annealing [20].

As regards STR typing, autosomal and Y-chromosome markers, all the selected tested samples exposed to salt and preserved at room temperature were successfully genotyped with a green flag for genotype quality status provided by the expert Genemapper-IDX software used. No allele dropout or amplification failure was observed in any of the samples in that group following manufacturer's instructions. Therefore, this storage method not only allows appropriate DNA recovery but also allows high-quality genetic profiles to be obtained, as qualified by the expert Genemapper-IDX software.

Surprisingly, in the case of room-temperature storage of untreated samples, a quantifiable DNA amount was obtained despite the storage time. This unexpected observation could be explained by the fact that the muscle samples were placed into sterile tubes that were kept sealed up to the moment of DNA extraction at the different time periods required. Under these conditions, the major degradation effect was endogenous with minimal, if any, microbial activity. A similar effect was also described by Campanella and coworkers, who found that buccal swabs from sunfish stored in the dark at room temperature for over 200 days could still yield DNA suitable for PCR amplification and DNA polymorphism detection [21]. However, in our assay, the quality of purified DNA from samples without treatment showed clear degradation reflected by the presence of poor amplification of the highest molecular weight loci or low relative fluorescent units (RFUs), which was not sufficient for assignment by the same analytical method used in the salt-preserved samples.

Availability, cost efficiency, experimental simplicity, undisturbed downstream processing, high quality of the genetic profiles obtained, as well as remarkable reduction of offending odours, are the principal advantages of the storage method proposed.

## Conclusions

Table salt preservation is an easy, economical and effective method when refrigeration is not immediately available or where sample transportation may be necessary. This technique should be considered as an additional method for sample storage when preservation of DNA integrity is required for PCR-based DNA typing.

## Materials and methods

### Tissue samples

Samples were maintained at room temperature in 2 ml polypropylene tubes (Axygen, Union City, California, USA), and kept closed up to the moment of DNA extraction. Fresh human psoas fragments of samples of 200 mg ± 20 mg were cut and exposed to three environmental conditions, with: (a) 1.5 g of table salt, mostly NaCl (Celusal, Buenos Aires, Argentina), (b) 1.5 g of garden soil and (c) no additional substances. DNA extraction from each sample was performed after 2, 4, 8 and 15 days and once a month for 1 year from the beginning of the experiment. Day 0 was considered the same day as death. Three samples per point were analysed. This tissue was chosen since muscle is a dominant soft tissue type throughout the body and is easily identifiable in fragmented body parts. In routine casework, muscle samples of 1 × 1 × 2 cm are placed into 50 ml polypropylene tubes previously filled with approximately half a volume of solid table salt.

### DNA extraction

All samples exposed to the diverse conditions tested (approximately 200 mg) were submitted to the extraction protocol consisting of a 5-min distilled water wash, overnight incubation at 56°C with 1 ml of TEC (Tris/ClH 10 mM pH 7.6 (Promega Corp., Madison, WI, USA), ethylenediaminetetra-acetic acid (EDTA) 1 mM pH 8 (Promega Corp., Madison, WI, USA), NaCl 100 mM (Carlo Erba, Milan, Italy) and 2% SDS (Life Technologies, Foster City, CA, USA) and 40 μl of 200 mg/ml proteinase K (Promega Corp., Madison, WI, USA). Two organic extractions, saturated phenol (Life Technologies, Foster City, CA, USA) and water saturated chloroform:isoamyl alcohol (Life Technologies, Foster City, CA, USA) (24:1) were performed and Microcon YM-100 (Milipore, Billerica, MA, USA) was used for purification and concentration of the extracted DNA. In routine casework, 100 mg of muscle tissue is cut from the inner part of the preserved sample and submitted to DNA extraction.

### DNA quantification

This was performed using Plexor HY (Promega Corp., Madison, WI, USA) by real-time PCR on a Corbett Rotor Gene 6000 (Corbett, Sydney, Australia) in a total reaction volume of 10 μl. The data have been normalised by dividing the total DNA detected (ng) by the amount of tissue (mg) of each sample.

### Statistical analysis

The statistical analysis (boxplot graph) was performed using Infosat 2.0 software [22].

### Genotype, haplotype and SNP typing

STR typing was performed by PowerPlex16 (Promega Corp., Madison, WI, USA) for autosomal markers. Standard conditions including 30 PCR cycles were performed for both kits following the manufacturer's instructions. PCR products were separated and visualised on an Applied Biosystems 3100 Avant Genetic Analyzer, and analysed with the expert Genemapper IDX software version 1.0 (Applied Biosystems, Foster City, CA, USA). Cutoff values of 50 RFU were used. The quality values of the allelic assignment are displayed as colour-coded flags: a green flag indicated that the sample passed the defined range for sizing quality (>0.75), a red flag indicated that the sample had a low quality range (<0.15) and, a yellow flag showed that the value between both ranges was attained. Mitochondrial and Y-chromosome haplogroups were tested by means of a multiplex real-time PCR followed by high resolution melting analysis [23].

### Analytical strategy

All the samples were quantitated by real-time PCR. A selected group of samples (time 0, room temperature samples with and without exposure to salt, samples stored for 30, 120 and 365 days, as well as a sample stored at -20°C for 365 days) were tested for autosomal and Y-chromosome STRs using commercial kits (Promega Corp., Madison, WI, USA) and mtDNA and Y-chromosome haplogroups. Since quantification results showed that no DNA was recovered from room temperature samples exposed to garden soil, none of them were genotyped.

## Competing interests

The authors declare that they have no competing interests.

## Authors' contributions

MC carried out experimental work, participated in the study design and wrote the manuscript. LB selected and provided biological samples and participated in the study design. DC designed and coordinated the study, wrote parts of the text and revised the final version of the manuscript. All authors read and approved the final manuscript.
